# Association of socio-economic environment and women’s empowerment with daily fruit and vegetable intake in Latin American cities: a multilevel study

**DOI:** 10.1186/s12889-025-22973-0

**Published:** 2025-07-02

**Authors:** Giovanna Valentino, Amy H. Auchincloss, Natalia Tumas, Nancy López-Olmedo, Ana Ortigoza, Mariana Carvalho de Menezes, Mónica Mazariegos, Carolina Nazzal

**Affiliations:** 1 Programa de Doctorado, Facultad de Medicina, Escuela de Salud Pública, Universidad de Chile, Santiago, Chile; 2 Departamento de Nutrición y Dietética, Escuela de Ciencias de la Salud, Facultad de Medicina, Pontificia Universidad Católica de Chile, Santiago, Chile; 3 Department of Epidemiology and Biostatistics, Dornsife School of Public Health, Drexel University, Philadelphia, PA USA; 4 Urban Health Collaborative, Dornsife School of Public Health, Drexel University, Philadelphia, PA USA; 5 Center of Research and Studies on Culture and Society, National and Technical Research Council and National University of Córdoba (CIECS, CONICET and UNC), Córdoba, Argentina; 6 Faculty of Medical Sciences, National University of Córdoba, Córdoba, Argentina; 7 Johns Hopkins University—Universitat Pompeu Fabra Public Policy (JHU-UPF PPC), Universitat Pompeu Fabra (UPF) - UPF Barcelona School of Management (UPF-BSM), Barcelona, Spain; 8 Center for Population Health Research, National Institute of Public Health, Cuernavaca, Mexico; 9 Department of Social and Environmental Determinants for Heath Equity, Pan American Health Organization, Washington DC, USA; 10 Department of Nutrition, Federal University of Minas Gerais, Belo Horizonte, Brazil; 11 INCAP Research Center for the Prevention of Chronic Diseases (CIIPEC), Institute of Nutrition of Central America and Panama (INCAP), Guatemala City, Guatemala; 12 Escuela de Salud Pública, Facultad de Medicina, Universidad de Chile, Independencia 939, Santiago, Chile

**Keywords:** Middle-income countries, Social determinants of health, Women’s empowerment, Diet quality, Socioeconomic development, Gross domestic income

## Abstract

**Background:**

In Latin America, a low proportion of the population meets the recommended fruit and vegetable (F&V) intake. The region is highly urbanized, with significant socioeconomic inequalities. The objective of this study was to analyze the association of the socio-economic environment (GDP per capita, living conditions) and women's empowerment (WE) with daily F&V intake in adults from Latin American cities, and whether these associations vary by individual education.

**Methods:**

Cross-sectional study using secondary data from the SALURBAL project (Urban Health in Latin America), which compiled data from health surveys, censuses, and other surveys from cities with ≥ 100,000 inhabitants in 11 Latin American countries. The sample included 91,977 adults from 234 cities in 8 countries with self-reported F&V intake data. The outcome was daily F&V intake, defined as consuming F&V 7 days a week. City-level exposures included GDP per capita (tertiles), living conditions score (overcrowding, piped water, and education access; Z-scores), and WE score (labor force participation and early marriage; Z-scores). We estimated prevalence ratios (PR) for the association between each exposure and daily F&V intake using gender-stratified two-level Poisson models with random intercepts for cities adjusted by city and individual-level covariates. Interaction terms were included to evaluate effect-modification by individual education.

**Results:**

Participants from cities in the upper GDP tertiles (T2 and T3) showed a ~ 7% higher prevalence of daily F&V intake among men (T2 PR = 1.08, 95% CI: 1.00–1.15; T3 PR = 1.06, 95% CI: 0.99–1.14) and women (T2 PR = 1.07, 95% CI: 1.01–1.13; T3 PR = 1.06, 95% CI: 0.99–1.12). A one standard deviation increase in WE and living conditions scores was associated with a ~ 10% higher prevalence of daily F&V intake in men (WE PR = 1.10, 95% CI: 1.02–1.19; living conditions PR = 1.10, 95% CI: 1.04–1.16) and women (WE PR = 1.11, 95% CI: 1.04–1.17; living conditions PR = 1.10, 95% CI: 1.05–1.15). Individual education levels significantly modified these associations (*p* < 0.05), which were stronger among those with lower educational attainment.

**Conclusions:**

City GDP per capita, living conditions and WE were directly associated with fruit and vegetable daily intake, particularly among individuals with lower education levels in Latin America.

**Supplementary Information:**

The online version contains supplementary material available at 10.1186/s12889-025-22973-0.

## Introduction

Globally, dietary risks account for 22% of deaths (~ 11 million) and 15% of disability-adjusted life years (~ 250 million DALYs) [1]. Diets low in fruits and vegetables are among the five most important dietary risks worldwide and in Latin America [1, 2]. In Latin America, around 7% of the population meets the World Health Organization recommendation for fruits and vegetables (F&V) intake (400 g/day), which varies among countries [3]. Healthy diets include a high proportion of F&V and are more sustainable for the environment, but affordability in Latin America is low, with approximately 22% of its population not able to afford a healthy food basket [4].

Age, gender, education, and socioeconomic status are important individual sociodemographic factors influencing access, choice, preparation, and food consumption [5–7]. Also, Latin America is a highly urbanized region, and urban social environment factors (e.g., living conditions, gender inequality, access to education, access to the labor market, and socioeconomic development) might also affect access, choice, and F&V intake. A few studies have shown that country-level GDP per capita, low inequality, and employment have been linked to higher intake of F&V and food security, likely due to their impact on food trade, investment, and access and affordability [5, 8–10]. Moreover, women’s empowerment can lead to changes in F&V intake and diet quality in two directions: higher power of decision-making and purchases, but also less time for cooking homemade meals (a domestic labor traditionally assigned to women) [11, 12]. Evidence from Sub-Saharan Africa, Bangladesh, and China indicates that women´s empowerment is linked to greater dietary diversity and household food security [13–16], particularly in the context of maternal and child nutrition. However, to the best of our knowledge, there is still lack of evidence in urban contexts of other regions.

Very little work has examined city-level social factors and dietary outcomes, with the exception of a descriptive ecological study that found higher prevalences of F&V daily intake in Latin American cities with more favorable socio-economic factors and women´s empowerment [17]. A previous multilevel study in Latin American cities reported a lower prevalence of excess weight (overweight/obesity) in women residing in cities with higher women’s empowerment [18]. Furthermore, the literature suggests that individual education and gender are effect modifiers in associations of women’s empowerment and other city-level variables with obesity and hypertension [18–20]. F&V intake is a modifiable behavior that can influence obesity and other health-related outcomes (e.g. hypertension), potentially showing shorter-term responses to public health interventions [21]. Therefore, understanding its association with the city social environment, including women´s empowerment, is crucial to design effective interventions in a region characterized by significant socioeconomic inequities. This study aimed to analyze the association of socioeconomic environment factors (GDP per capita and living conditions) and women’s empowerment with daily intake of F&V in adults residing in Latin American cities and to assess whether these associations vary by individual education.

## Methods

### Design and sample

**Fig. 1:**
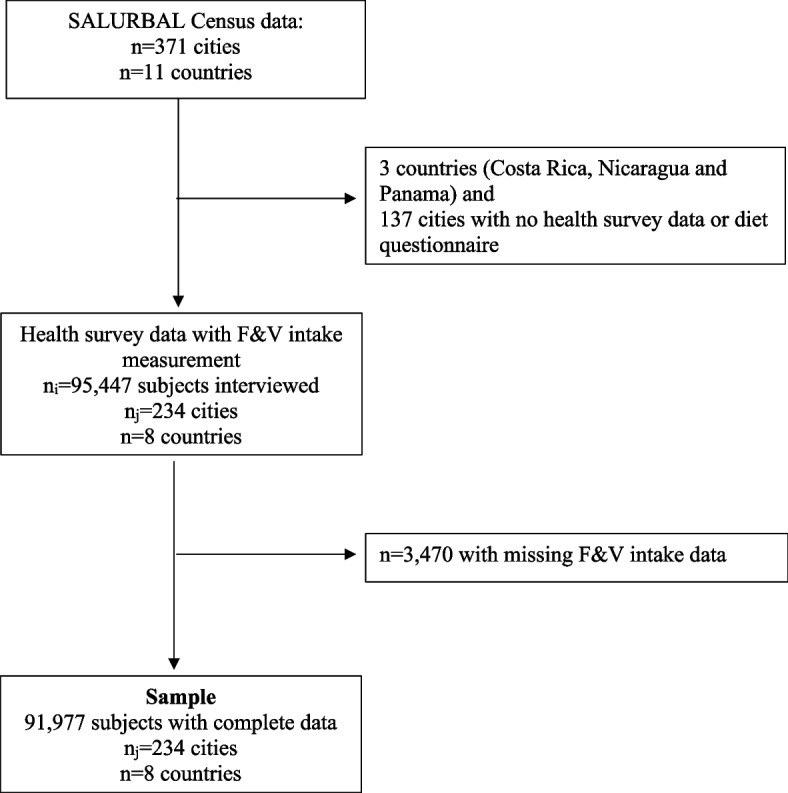
Sample flowchart

This cross-sectional study used secondary data from the SALURBAL project (Salud Urbana en América Latina—Urban Health in Latin America) [22]. SALURBAL compiled and harmonized databases of health surveys, census, and other surveys from 371 cities with ≥ 100,000 inhabitants from 11 Latin American countries (further detailed elsewhere [22]). Dietary data came from adult survey participants (18–97 years old; *N* = 91,977) in 234 cities from the 8 SALURBAL countries that have self-reported intake of F&V from health surveys: Argentina 2013, Brazil 2013, Chile 2017, Colombia 2015, El Salvador 2014, Guatemala 2002, Mexico 2018, and Peru 2016 (Fig. 1). National health surveys of each country provided geographic identifiers for the region/state and city or municipality of the participants'residence. SALURBAL selected participants whose geographic identifiers matched to the cities selected in SALURBAL [22]. This provided a sample of survey participants in urban areas [22].

### Outcome: daily fruit and vegetable intake

Most countries asked for frequency intake of each group (fruits and vegetables), which were then harmonized by SALURBAL (for detailed information of the original questionnaires and the harmonization process, see Table S1) [22]. The frequency of fruit and vegetable intake (days/week) was summed to classify whether fruits and/or vegetables were consumed 7 days a week. The variable was defined as a binary outcome (daily intake vs. non-daily intake).

### Exposure: city socio-economic factors and women’s empowerment

City-level social environment variables came from the closest available year to the health survey data of each country (Table S2). City-level per capita Gross Domestic Product (GDP) was obtained from a gridded global dataset of previous estimations for subnational data for each country (expressed in constant 2011 USD purchase power parity) and was categorized in tertiles [23–25]. Data was available yearly from 1990 to 2015, so the year was matched with the same year in which F&V data was collected, except Chile, Mexico, and Peru, where the year 2015 was used.

Table S3 shows the components that were used to derive city-level living conditions score and women’s empowerment. In brief, city-level living conditions was proxied by a socioeconomic score derived through principal component analysis, based on three census variables: 1) the proportion of households with access to piped water inside dwelling, 2) the proportion of households that are not overcrowded (< 3 people per room), and 3) the percentage of the population between 15 and 17 years old enrolled in the education system [26]. The three variables were subsequently transformed into Z-scores and summed.

City-level women’s empowerment (WE) was proxied by women´s achievement score for autonomy (previously described elsewhere [27]) calculated using two census variables selected from factor analyses: female labor force participation and formal marriage in women between 15 and 17 years old [27]. These two variables were subsequently transformed into Z-scores and summed [27].

Each of the summation scores (living conditions and WE) was standardized to a mean of 0 and a standard deviation (SD) of 1 based on the distribution of scores across the sample of cities. Higher values indicate higher socioeconomic development and women’s empowerment (Table S3).

### Covariates

Covariates were identified a priori via scientific literature, and then we used a Directed Acyclic Graph (DAG) to verify that each variable was appropriate to include in the model [28]. We included individual-level covariates from health surveys, *age and educational level.* All these variables have been previously associated with F&V intake and dietary choices [5–8, 29]. Individual-level education was self-reported for the highest completed grade or qualification and was harmonized and categorized according to IPUMS criteria as follows [22]: 1) Less than primary, 2) Primary, 3) Secondary, or 4) University (harmonization details in Table S4). City-level covariates considered for model adjustment were *country, city population size, climate zone, city educational attainment, and city-level per capita GDP (in models in which GDP was not the exposure).* City population size was defined as the population projection in the city administrative area for the year closest to the country’s national health survey. It was included due to prior evidence showing associations between the population size of the residential area and the F&V intake [30]. The climate zone was classified using the Köppen system, which considers four main categories: tropical, arid, temperate, and polar [31, 32]. It was included because the climate zone affects F&V local availability, varies between cities within most countries, and has been previously associated with economic growth (seasonality from temperate weather forced higher human capital and technological development) [33]. City *educational level achievement* was proxied as the proportion of the population aged 25 years or older with high school education at the city level. GDP per capita and population educational attainment were considered as covariates because they are closely related to human capital, women’s labor force participation, and access to better living conditions [29, 34].

### Effect modifiers

Considering that prior work reported that the association between social indicators and nutrition-related conditions differed by gender [18–20], we stratified the analysis by gender (using self-reported sex as female/male). Additionally, we considered individual-level education as a potential effect modifier, as these studies suggest that the associations also vary across individual education levels [18–20].

### Statistical analysis

We first described the individual and contextual characteristics of the sample. We compared differences between daily consumers and non-daily consumers of F&V using Mann–Whitney and Chi-square tests. Then, we estimated the association between social environment factors and individual daily F&V intake using gender-stratified two-level Poisson models, with a random intercept for cities. Models of increasing complexity were explored. First, we tested a null model with country as fixed effects and a random variance component for city level. Model 1 (minimally adjusted model) included each city exposure variable in separate models (Model A = GDP per capita; Model B = Women’s empowerment; Model C = Living conditions score). Model 2 added in city and individual-level covariates. Finally, we added interaction terms to evaluate whether the associations between city-level social environment factors and daily F&V intake were modified by individual education.

$$\begin{array}{c}\log\left(\lambda ij\right)=\;\beta_{0ij}\;+\;\beta_1Country_j\;+\;\beta_2Exp_{j\;}+\;\beta_3CZ_j\;+\beta_4Educ_j\;+\;\beta_5GDP_j+\beta_6Pop_j\;+\beta_7Age_{ij}\;+\;\beta_8IndE_{ij}\\+\beta_9Exp_{j\ast}IndE_{ij}\\\beta_{0ij}\;=\;\gamma_{00}\;+v_{oj}\\\lambda_{ij}\sim Y_{ij}\end{array}$$

Where *λ*_*ij*_ is the expected prevalence of F&V daily intake conditioned by city and individual-level predictors; *β*_*0ij*_ is the random intercept considering city-level residuals (*v*_*0j*_); *β*_*1*_ is the fixed effect of the country on F&V intake; *β*_*2*_ is the effect of each city-level exposure variable (*Exp*_*j*_: Model A = GDP per capita, Model B = women’s empowerment, Model C = living conditions score); *β*_*3*_ = is the effect of city climate zone [CZ_*j*_]; *β*_*4*_ = is the effect of city educational attainment [Educ_*j*_]; *β*_*5*_ = is the effect of GDP when used as covariate in model B and C [GDP_*j*_];* β*_*6*_ is the effect of city size [Pop_j_]; *β*_*7*_ is the effect of age; *β*_*8*_ is the effect of individual educational level; *β*_*9*_ is the interaction effect between the city-level exposure and individual educational level. Exponentiated coefficients indicate prevalence ratios for F&V daily intake.

Missing values for individual educational level (*N* = 106; 0.1% of the sample) were kept in all analyses as an independent category. We estimated that our sample size provided at least 80% power to detect significant effects for prevalence ratios of 1.07 or higher, or 0.93 or lower. This estimation assumes three tertiles of 78 cities, with an average sample size of 410 adults per city and an intraclass correlation coefficient of 0.04 (adjusted by country). All analyses were performed using Stata 18.0 [35].

### Sensitivity analyses

Our primary analysis uses a combined outcome for fruits and/or vegetables because that operationalization aligns with WHO and the region’s dietary recommendations. In supplementary sensitivity analyses, we used two alternate disaggregated outcomes: 1) daily intake of fruits, and 2) daily intake of vegetables.

## Results

Out of 91,977 surveyed adults, 63.6% have daily F&V intake. Among those with daily intake, 50% consumed fruits and 52% vegetables daily, whereas 27% consumed both of them (*N* = 15,496). Table 1 describes the individual and contextual characteristics of the sample. Daily consumers reside in cities with higher women’s empowerment (WE, media*n Z-score* = 1.07 vs. 1.00), higher GDP (median USD = $15,624 vs. $13,297), and better living conditions (median Z-score = 0.91 vs. 0.45) (*p* < 0,05).

**Table 1:** Sociodemographic and contextual characteristics of the sample according to daily intake of fruits and vegetables

	**Total *N* = 91,977**	**Daily intake *N* = 58,496**	**Non-daily intake *N* = 33,481**
Daily intake of fruits, n (%)	29,470 (32.0%)	29,470 (50.4%)	0 (0%)
Daily intake of vegetables, n (%)	30,242 (32.9%)	30,242 (51.7%)	0 (0%)
Mean fruit & vegetable intake, servings/day^a^	1.7 (1.0; 3.0)	2.1 (1.4; 3.0)	0.9 (0.6; 1.4)
Age, years	40 (29; 55)	42 (31; 57)	37 (27; 51)
**Gender**			
Women, n (%)	53,433 (58.1%)	36,072 (61.7%)	17,361 (51.9%)
Men, n (%)	38,544 (41.9%)	22,424 (38.3%)	16,120 (48.2%)
**Educational level**			
Not reported, n(%)	106 (0.1%)	53 (0.1%)	53 (0.2%)
Less than primary, n (%)	16,022 (17.4%)	9,499 (16.2%)	6,523 (19.5%)
Primary, n (%)	28,993 (31.5%)	16,951 (29.0%)	12,042 (36.0%)
High school, n (%)	34,068 (37.0%)	22,234 (38.0%)	11,834 (35.4%)
University, n (%)	12,788 (13.9%)	9,759 (16.7%)	3,029 (9.1%)
**City characteristics**			
City size (persons per 100,000)	11.1 (4.3; 35.7)	11.8 (4.4; 36.8)	10.3 (3.7; 33.5)
WE, Z-Score	1.06 (0.49; 1.45)	1.07 (0.64; 1.46)	1.00 (0.18; 1.49)
GDP per capita, USD 2011 ppp	14,487 (9,429; 22,036)	15,624 (10,070; 22,186)	13,297 (9,095; 21,808)
Living conditions score, Z-Score	0.76 (0.07; 1.18)	0.91 (0.22; 1.19)	0.45 (−0.16; 1.01)
Population with high school education, %	46.0 (40.0; 52.1)	44.9 (40.0; 51.2)	47.0 (40.7; 54.7)
**Climate Zone**			
Tropical, n (%)	38,420 (41.8%)	24,391 (41.7%)	14,029 (41.9%)
Arid, n (%)	20,497 (22.3%)	11,310 (19.3%)	9,187 (27.4%)
Temperate, n (%)	31,704 (34.5%)	21,863 (37.4%)	9,841 (29.4%)
Polar, n (%)	1,356 (1.5%)	932 (1.6%)	424 (1.3%)
**Countries**			
Argentina, n (%)	21,261 (23.1%)	14,725 (25.2%)	6,536 (19.5%)
Brazil, n (%)	37,621 (40.9%)	26,295 (45.0%)	11,326 (30.1%)
Chile, n (%)	3,805 (4.1%)	2,999 (5.1%)	806 (2.4%)
Colombia, n (%)	4,433 (4.8%)	2,238 (3.8%)	2,195 (6.6%)
Guatemala, n (%)	1,396 (1.5%)	896 (1.5%)	500 (1.5%)
Mexico, n (%)	10,002 (10.9%)	4,199 (7.2%)	5,803 (17.3%)
Perú, n (%)	11,920 (13.0%)	6,389 (10.9%)	5,531 (16.5%)
El Salvador, n (%)	1,539 (1.7%)	755 (1.3%)	784 (2.3%)

Numeric variables are expressed in median (p25-p75), and categorical variables in absolute and relative frequency

*WE* Women’s empowerment, *GDP* Gross domestic product, *ppp* purchasing power parity

^a^Servings/day is not comparable between countries. All *p* values were < 0.005 when testing row differences for daily vs non-daily intake, except for tropical climate zone (*p* > 0.7) and Guatemala (*p* > 0.6). (Chi^2^ or Mann Whitney tests). City GDP per capita expressed in 2011 USD power purchase parity (ppp). Women’s empowerment was proxied from women autonomy score (Z-Score), which included female labor force and proportion of female population with formal marriage between 15–17 years old. Living conditions score (Z-Score) was proxied by living conditions score, which included proportion of households with piped water inside dwelling, proportion of households with overcrowding and proportion of population aged 15 to 17 years old inserted in the educational system

### Associations with city socio-economic environment and women’s empowerment

Table 2 shows the minimally adjusted (model 1) and fully adjusted (model 2) associations between each social environment exposure (GDP, WE, living conditions score) and daily F&V intake in men and women. After adjusting for individual and city covariates (Model 2), compared to the lowest level of city GDP (tertile 1), survey participants living in cities with mid-level GDP (tertile 2) had ~ 7% higher prevalence of daily F&V intake (PR = 1.07 [95% CI: 1.01, 1.13] for women, and PR = 1.08 [95% CI: 1.00, 1.15] for men). Similar point estimates for daily intake were observed for the highest tertile of city GDP (vs. lowest), although it was not statistically significant (PR = 1.06 [95% CI: 0.99, 1.12] for women, and PR = 1.06 [95% CI: 0.99, 1.14] for men).

**Table 2:** Gender-stratified prevalence ratios of fruits and vegetables daily intake associated with city social environment variables

City contextual characteristics	Women (*N* = 53.433)		Men (*N* = 38.544)	
	Model 1 PR (95% CI)	Model 2 PR (95% CI)	Model 1 PR (95% CI)	Model 2 PR (95% CI)
**A. Gross domestic product, per capita**				
Tertile 1 (< $11,100 USD ppp)	Reference	Reference	Reference	Reference
Tertile 2 ($11,100 to $16,200 USD ppp)	1.08 (1.02, 1.14)^*^	1.07 (1.01, 1.13)^*^	1.08 (1.00, 1.16)^*^	1.08 (1.00, 1.15)^*^
Tertile 3 (> $16,200 USD ppp)	1.07 (1.01, 1.13)^*^	1.06 (0.99, 1.12)	1.06 (0.99, 1.13)	1.06 (0.99, 1.14)
**B. Women’s empowerment**				
Z-Score, each 1 SD increase	1.12 (1.06, 1.17)^**^	1.11 (1.04, 1.17)^*^	1.09 (1.02, 1.17)^*^	1.10 (1.02, 1.19)^*^
**C. Living conditions score**				
Z-Score, each 1SD increase	1.10 (1.05, 1.14)^**^	1.10 (1.05, 1.15)^**^	1.08 (1.02, 1.14)^*^	1.10 (1.04, 1.16)^*^

City GDP per capita expressed in 2011 USD power purchase parity (ppp). Women’s empowerment was proxied from women autonomy score (Z-Score), which included female labor force and proportion of female population with formal marriage between age 15–17 years old. Living conditions score included proportion of households with piped water inside dwelling, proportion of households with overcrowding and proportion of population aged 15 to 17 years old inserted in the educational system

Model 1: two-level Poisson regression for each exposure with city as random intercept and country as fixed effects

Model 2: used the structure from model 1 and adjusted for city size (population), city climate zone, city educational attainment, individual age and individual educational level. In exposure “B” and “C”, city GDP was also adjusted for

*PR* Prevalence Ratio, *CI* Confidence Interval, *ppp* purchasing power parity

^*^p < 0.05

^**^p < 0.001

In addition, a one standard deviation increase in living conditions was associated with approximately 10% higher prevalence of daily intake of F&V in both women (PR = 1.10 [95% CI 1.05, 1.15]) and men (PR = 1.10 [95% CI: 1.04, 1.16]).

Finally, a one standard deviation in women’s empowerment also associated with ~ 10% higher prevalence of daily F&V intake in women (PR = 1.11 [95% CI: 1.04, 1.17]) and men (PR = 1.10 [95% CI: 1.02, 1.19]; Table 2).

### Effect modification by individual education level

**Fig. 2:**
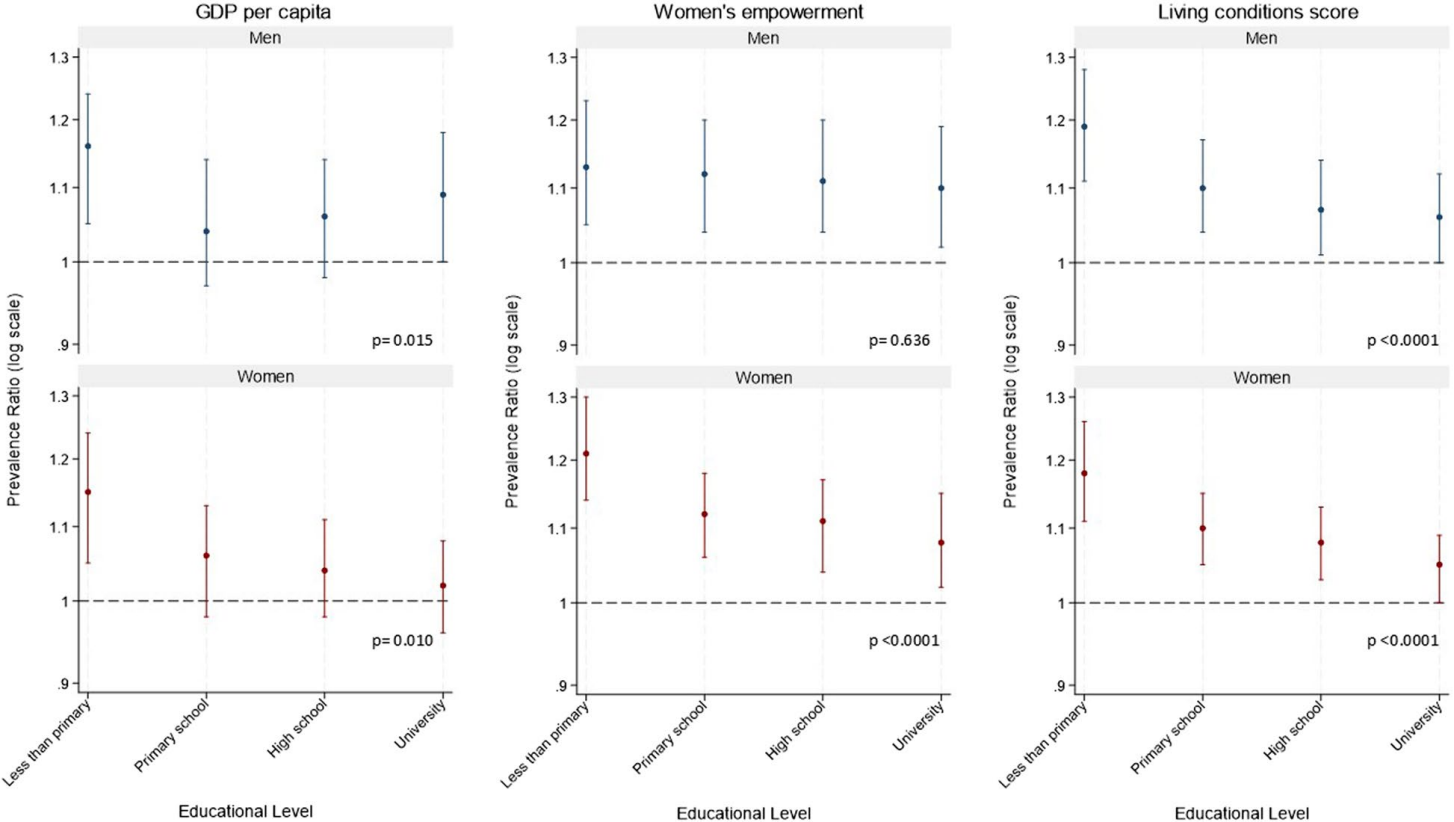
Gender-stratified prevalence ratios of F&V daily intake associated with city variables by individual-level education. GDP: Gross Domestic Product. *P* values indicate the global *p* value for the interaction between each social environment exposure and individual educational level in men and women for daily intake of F&V. GDP per capita show prevalence ratios for Tertile 3 vs. Tertile 1. Women’s empowerment and Living conditions score show prevalence ratios for each 1 standard deviation increase on each index

Figure 2 shows the associations between the daily intake of F&V and the exposures by categories of individual education in men and women. The global *p*-value for interaction was significant for living conditions (*p* < 0.0001) and GDP per capita (Tertile 1 vs. Tertile 3, *p* < 0.05) in both genders, and for WE only in women (*p* < 0.0001). Overall, the associations between the city social environment and F&V intake were strongest (most apparent) among individuals with less than primary education (Fig. 2 estimates are detailed in Table S5).

### Sensitivity analyses

Compared to the main analyses, when the outcome was operationalized as daily intake of *fruits* and daily intake of *vegetables* (disaggregated outcome, Table S6), the magnitude of some associations showed noteworthy differences. First, the PRs between GDP per capita and daily intake of *fruits* showed a higher magnitude compared to using the combined outcome (GDP per capita tertile 3 vs tertile 1 PRs were 1.17 [95% CI: 1.07, 1.28] in women and 1.13 [95% CI: 1.01, 1.26] in men vs. the combined outcome PR = 1.06 [95% CI: 0.99, 1.14] in both genders). Second, although PRs between the highest tertile of GDP per capita and daily intake of vegetables were also positive and similar to the main outcome, they did not reach statistical significance (women: PR = 1.11 [95% CI: 0.97, 1.27]; men: PR = 1.09 [95% CI: 0.93, 1.28]). Third, PRs between WE and daily intake of *vegetables* showed a higher magnitude compared to using the combined outcome (PRs were 1.24 [95% CI: 1.10, 1.39] in women and 1.17 [95% CI: 1.02; 1.33] in men vs. the combined outcome PR = 1.11 [95% CI: 1.04, 1.17] in women and PR = 1.10 [95% CI: 1.02, 1.19] in men). Fourth, although WE was positively associated with daily fruit intake in women (PR = 1.08 [95% CI: 0.99, 1.17]), no effect was observed for men (PR = 1.00 [95% CI: 0.89, 1.13]). Similar results for interaction terms were observed compared to the main analysis with stronger associations between each exposure and daily intake of fruits or vegetables at the lowest educational level (stratified results and interaction *p*-values reported in Table S7 and Table S8).

## Discussion

This is the first study to examine the association of city socioeconomic factors and women´s empowerment with individual intake of F&V in Latin America. We found that higher women’s empowerment, higher per capita GDP, and better living conditions at the city level were associated with higher prevalences of daily F&V intake. The associations were modified by individual education; in general, the effect of contextual variables on F&V intake was stronger among individuals with less than primary education, representing the lowest education level. Moreover, we found some heterogeneity in the associations when analyzing the daily intake of fruits and vegetables as separate outcomes.

### Association of F&V daily intake with city GDP and living conditions score

In our study, we found a positive association between city per capita GDP and the prevalence of F&V daily intake, and this association was stronger for daily intake of fruits. Our results may be due to higher GDP being correlated in Latin American contexts with higher affordability and availability of F&V, which could result in higher purchases and intake [5, 8, 9]. While our study context, unit of analysis (cities), and design do not fully match the extant literature, our findings align with the work reported by a few studies. Extant research found that country-income/GDP was associated with a higher intake of fruits and vegetables in lower and higher-income contexts across diverse regions [5, 8, 36]. Similar to previous research, we found a threshold for the positive association observed between GDP per capita and F&V intake, in which no further strength was observed in the upper tertile (USD > 16,000) when compared to the intermediate tertile (USD 11,200 to 16,200) [36]. This suggests that improvements in GDP per capita may play a more significant role among low-income contexts or countries. Finally, in the sensitivity analysis, we found a stronger association of GDP with daily fruit intake, but not a significant association for vegetable intake. Work from Latin America (Chile) found household-level income was associated with household fruit purchases but not vegetable purchases, which aligns with our results [37]. The authors attributed this to higher economic costs and fewer barriers related to cooking skills for fruit consumption [37].

We used a metric of city socioeconomic level based on living conditions: proportion of households with piped water, not overcrowded, and with higher access to education [26]. We found that the city living conditions score was positively associated with daily F&V intake, even after adjusting for other individual and city-level characteristics (such as gender, age, educational level, and city-GDP, among others). This association might reflect access to better city- and household-level amenities, such as higher availability of F&V retail stores and healthier food environments in the neighborhoods within cities with higher socioeconomic development [38, 39], as well as household amenities that ensure safe preparation and storage of F&V (e.g., electricity, refrigeration, piped water). Although there is limited evidence regarding our city-level socioeconomic index and F&V intake, previous evidence has shown similar associations between neighborhood socioeconomic status (SES) and F&V intake (or biochemical markers of F&V intake) [38, 40–42].

### Association of F&V daily intake with women’s empowerment

A higher prevalence of daily intake of F&V in both genders was observed in cities with higher WE. Among possible mechanisms explaining this association is that higher participation of women in the labor force increases the purchasing power of households and the power of decision-making by women, who usually purchase healthier food [37, 43]. Indeed, globally, on most survey data, women have a higher intake and purchase of F&V than men [5, 6, 37, 43, 44]. This difference may be attributed to gender roles that persist even among women who participate in the workforce (unpaid reproductive domestic work traditionally assigned to women, along with higher pressure to align to hegemonic body size) [45, 46]. In line with that, women are more prone to attending health controls at earlier ages, which influences health awareness and the behaviors of their families [47]. Moreover, we found that WE had a stronger association with daily vegetable intake in both genders. Unlike most fruits, many vegetables tend to require more time for preparation and/or cooking skills. Therefore, the home food environment might be influenced by healthier culinary habits of empowered women, favoring vegetable consumption among all household members.

### Effect modification of individual education level

More favorable city contexts (GDP, WE, living conditions) appeared to be particularly beneficial for those with the lowest education. Individuals with higher levels of education may have higher levels of agency and resources to meet their needs despite of living in unhealthy food environments. A study from the EPIC-Norfolk cohort in the United Kingdom (2004) showed similar results: the effect of living in a deprived area on F&V intake was stronger in those groups with low-skilled manual occupation or who did not have formal education [48]. For WE, the interaction was evident with individual education for women but not for men. This suggests that men may benefit indirectly from the household food environment in cities with higher WE due to persistent gender roles across diverse educational levels, as previously explained.

Furthermore, we only found interaction with individual education in the association of the upper tertile of GDP per capita with daily F&V intake, but not the intermediate one. Previous country-level data showed that disparity in F&V intake according to the individual educational level was highest in low-middle-income countries and slightly decreased in upper-middle-income countries [5]. This might reflect the diffusion theory, which suggests that socioeconomic development initially affects the behaviors of early adopters (usually the most privileged in society), increasing inequality. However, as the majority adopt the behavior, inequality diminishes, potentially benefiting the least privileged at higher levels of development [49].

### Public health policy implications

Our results are relevant to public policy in several aspects. First, the results highlight how the city´s social environment is related to F&V intake, especially in the most vulnerable population – suggesting the role that city contexts can play in reducing individual-level health disparities. These results suggest that people with low educational levels are more context-sensitive and may benefit more from improvements in the urban social environment. Therefore, improving economic and physical access in populations at the lowest socioeconomic level might increase the intake of F&V (e.g., subsidies, food banks, improving the location, infrastructure, and density of street markets and retailers selling F&V) [50, 51]. Still, attention should also be paid to social policy that reduces material deprivation and improves living conditions of the urban population. Second, policies that reduce unemployment, improve work schedule flexibility, and favor access to universal daycare and paid maternal leave might increase women’s participation in the labor force and autonomy [46, 52], which may lead to higher household incomes and purchases of F&V. Third, these results highlight the need for strengthening urban food systems in Latin America to improve access to healthy diets across the population [50, 53].

### Strengths and limitations

The main strength of our study is that it is the first multilevel study using data from 234 cities in Latin America to estimate the association of the city’s social environment with F&V intake. These results help explain disparities in F&V intake within countries across the region. However, this study also has some limitations. First, we did not use compliance with the World Health Organization recommendations (≥ 5 servings/day) because servings were not possible to estimate precisely with all countries’ survey instruments. To reduce differential bias, we used daily intake of F&V (7 days a week). For this reason, we cannot ensure that those with daily intake meet the recommendation of at least one serving a day. However, this group had a significantly higher average intake (servings per day) when analyzing the subsample with these data (Table 1). In this study, we assumed that daily intake is the first step for meeting the WHO recommendation. Therefore, we would expect similar results if we used compliance of ≥ 5 servings/day, as previous country-level evidence has shown even wider differences in the same direction when comparing intake ≥ 5 servings/day by country-level GDP and individual sociodemographic characteristics [5]. Also, as with any cross-sectional study, we cannot establish the temporality between the data sources for exposures and outcomes. In a couple of countries (Chile and Colombia), there was a long lag between the living conditions census-derived exposure and survey-derived F&V intake; however, census data might take a long time to change, and we added country as a fixed effect in all the models to adjust for country differences. The associations between the upper tertile of GDP per capita and daily F&V consumption did not reach statistical significance in the main analysis; the power for this estimate was 68%, which limits the precision and increases the likelihood of a type II error. Moreover, we could not provide a stratified analyses by country due to lack of power for such analyses and the complexity added for interpreting the re-standardized indicators. Finally, we cannot rule out the possibility of selection bias due to: 1) non-response rates in some original health surveys [54–56]; and 2) differences between our sample and participants excluded (*N* = 3,470) due to missing outcome data (see Table S9). Most of the missing data originated from Brazil and participants resided in bigger cities with tropical weather. They were younger, more often male and had higher levels of education.

## Conclusion

Factors related to the city’s socio-economic factors and women’s empowerment are positively associated with individual F&V daily intake, particularly among populations with low educational levels. The findings underscore the importance of considering local policies and interventions regarding economic development, living conditions and women’s empowerment to improve diet quality in the Latin American region. Further research is needed to explore and develop targeted strategies to improve F&V intake in the region.

## Supplementary Information

Supplementary Material 1.

Supplementary Material 2.

Supplementary Material 3.

Supplementary Material 4.

Supplementary Material 5.

Supplementary Material 6.

Supplementary Material 7.

Supplementary Material 8.

Supplementary Material 9.

## Data Availability

The harmonized health surveys data that support the findings of this study were available from SALURBAL, but restrictions apply to the availability of these data, which were used under license for the current study, and so are not publicly available. Most of the datasets from city-level variables analyzed during the current study are publicly available at https://data.lacurbanhealth.org/. A link to the agency website can be accessed at https://drexel.edu/lac/data-evidence/data-acknowledgements/.
