# Supplementary material for: Association of socio-economic environment and women’s empowerment with daily fruit and vegetable intake in Latin American cities: a multilevel study
Source: BMC Public Health. 2025 Jul 2;25:2189. doi: 10.1186/s12889-025-22973-0 (PMC12219996; doi:10.1186/s12889-025-22973-0)
Supplement: Supplementary file 1 — Supplementary Material 1. [file 12889_2025_22973_MOESM1_ESM.docx]

**Table S1. Summary of the harmonization process of fruit and vegetable data from original health surveys**

|  | **Original Health Survey Questionnaire** | | **Harmonization process** | | |
| --- | --- | --- | --- | --- | --- |
|  | **Fruit intake frequency** | **Vegetable intake frequency** | **Fruit intake frequency** | **Vegetable intake frequency** | **F&V intake frequency** |
| **Argentina** | In a typical week, how many days do you eat fruits? Response: Number of days (Numeric), Never, Doesn’t know | In a typical week, how many days do you eat vegetables? Response: Number of days (Numeric), Never, Doesn’t know | 0-7 days | 0-7 days | Sum of Fruit intake frequency and Vegetable intake frequency capped to 7 days (Numeric: 0-7 days) |
| **Brazil** | How many days a week do you eat fruits?  Response: number of days | How many days of the Q1. How many days of the week do you eat lettuce and tomato salad or any other raw vegetable salad?; Q2. On how many days of the week do you usually eat cooked vegetables or vegetables such as cabbage, carrots, chayote, eggplant, zucchini? (not counting potatoes, cassava, or yams) Response: number of days | 0-7 days | 0-7 days of most frequent vegetable (raw or cooked) |  |
| **Chile** | In a typical week, how may days do you eat fruits? Response: number of days | In a typical week, how many days do you eat vegetables or vegetable salads? Don’t include potatoes or legumes. Response: number of days | 0-7 days | 0-7 days |  |
| **Colombia** | How often do you eat these food items? [whole fruits]. Responses: Less than once a month, once a month, 2-3 times per month, once a week, twice a week, three to four times a week, five to six times week, once a day, twice a day three times or more per day | Q1. How often do you eat these food items? [Cooked vegetables]. Q2. How often do you eat these food items?[Raw vegetables]. Responses (Q1 and Q2): Less than once a month, once a month, 2-3 times per month, once a week, twice a week, three to four times a week, five to six times week, once a day, twice a day three times or more per day | 0-7 days (ranges were averaged: 0, 0.5, 1, 2, 3.5, 5.5, 7) | 0-7 days of most frequent vegetable (raw or cooked) (ranges were averaged: 0, 0.5, 1,2,3.5,5.5, 7) |  |
| **El Salvador** | In a typical week, how many days do you eat fruits? Response: number of days, doesn’t know | In a typical week, how many days do you eat vegetables or vegetable salads? Response: number of days, doesn’t know | 0-7 days | 0-7 days |  |
| **Guatemala** | In the last month, have you eaten Fruits without counting juices? How much? (Frequency). Responses: Never, daily, once per week, twice per week, 3 times per week, 4 times per week, 5 times per week, 6 times per week, 1 time per month, 2 times per month, 3 times per month, Do not know | In the last month, have you eaten vegetables? How much? (Frequency). Responses: Never, daily, once per week, twice per week, 3 times per week, 4 times per week, 5 times per week, 6 times per week, 1 time per month, 2 times per month, 3 times per month, Do not know | 0-7 days (monthly frequency divided by 4: 0, 0.25, 0.5, 0.75, 1, 2, 3, 4,5,6, 7) | 0-7 days (monthly frequency divided by 4: 0, 0.25, 0.5, 0.75, 1, 2, 3, 4,5,6, 7) |  |
| **Mexico** | Full FFQ: In the last seven days…How many days did you eat (specific fruit)? (included 13 fresh fruit items) Response: 0 to 7 days | Full FFQ: In the last seven days...How many days did you eat (specific vegetable)? (included 17 vegetable items). Response: 0 to 7 days | 0-7 days of most frequent fruit | 0-7 days of most frequent vegetable: |  |
| **Peru** | In the past 7 days, or from last [date] until yesterday, how many days did you eat fruits? If you have eaten fruits. Response: number of days, did not eat, doesn’t know | In the past 7 days, or from last [date] until yesterday, how many days did you eat vegetables? If you have eaten vegetables. Response: number of days, did not eat, doesn’t know | 0-7 days | 0-7 days |  |
