# Supplementary material for: Association of socio-economic environment and women’s empowerment with daily fruit and vegetable intake in Latin American cities: a multilevel study
Source: BMC Public Health. 2025 Jul 2;25:2189. doi: 10.1186/s12889-025-22973-0 (PMC12219996; doi:10.1186/s12889-025-22973-0)
Supplement: Supplementary file 2 — Supplementary Material 2. [file 12889_2025_22973_MOESM2_ESM.docx]

**Table S2. Data availability for health surveys and sociodemographic variables**

| Country | National Health Survey | Census  (Living conditions and WE) | Subnational GDP |
| --- | --- | --- | --- |
| Argentina | 2013 | 2010 | 2013 |
| Brazil | 2013 | 2010 | 2013 |
| Chile | 2017 | 2017^ab^ | 2015 |
| Colombia | 2015 | 2018^c^ | 2015 |
| El Salvador | 2014 | 2007 | 2014 |
| Guatemala | 2002 | 2002 | 2002 |
| México | 2018 | 2020 | 2015 |
| Perú | 2016 | 2017 | 2015 |

WE: Women’s empowerment.  ^a^Early Marriage data from census 2002, ^b^Living conditions data from census 2002, ^c^Living conditions data from census 2005
