# Supplementary material for: Association of socio-economic environment and women’s empowerment with daily fruit and vegetable intake in Latin American cities: a multilevel study
Source: BMC Public Health. 2025 Jul 2;25:2189. doi: 10.1186/s12889-025-22973-0 (PMC12219996; doi:10.1186/s12889-025-22973-0)
Supplement: Supplementary file 3 — Supplementary Material 3. [file 12889_2025_22973_MOESM3_ESM.docx]

**Table S3 Socioeconomic characteristics of the 234 Latin American cities according to tertiles for the socioeconomic score of living conditions and the women’s empowerment score built by SALURBAL**

|  | **Socioeconomic score of living conditions (Z-scores)** | | |
| --- | --- | --- | --- |
|  | **Tertile 1 (<-0.23)** | **Tertile 2 (-0.23 to 0.56)** | **Tertile 3 (>0.56)** |
| ***Households with piped water inside^b,c^, %*** | ***74.5 (11.7)*** | ***89.0 (6.8)*** | ***94.6 (3.1)*** |
| ***Households without overcrowding (<3 people/room)^b^, %*** | ***90.7 (4.1)*** | ***94.9 (2.7)*** | ***96.6 (1.4)*** |
| ***Population aged 15-17 years in the educational system, %*** | ***74.6 (7.1)*** | ***80.4 (5.4)*** | ***87.1 (5.3)*** |
|  | **Women empowerment score for autonomy (Z-scores)^b^** | | |
|  | **Tertile 1 (<-0.50)** | **Tertile 2 (-0.50 to 0.49)** | **Tertile 3 (>0.49)** |
| ***Female labor force, %*** | ***43.5 (4.2)*** | ***46.5 (3.90)*** | ***56.0 (4.7)*** |
| ***Early marriage (proportion of married women aged 15-17 years)^b^, %*** | ***2.4 (1.4)*** | ***0.9 (0.6)*** | ***0.2 (0.2)*** |

Variables in cursive and bold format are the ones included in each indicator. Data expressed in mean and standard deviation. Data from census of Argentina (2010), Brasil (2010), Chile (2017), Colombia (2018), El Salvador (2007), México (2020), Perú (2017) and Guatemala (2002), unless otherwise is indicated. ^a^187 cities with data. ^b^ Data for Chile 2002, ^c^Data for Colombia 2005, ^d^Does not include data for Brazil.
