# Supplementary material for: Association of socio-economic environment and women’s empowerment with daily fruit and vegetable intake in Latin American cities: a multilevel study
Source: BMC Public Health. 2025 Jul 2;25:2189. doi: 10.1186/s12889-025-22973-0 (PMC12219996; doi:10.1186/s12889-025-22973-0)
Supplement: Supplementary file 4 — Supplementary Material 4. [file 12889_2025_22973_MOESM4_ESM.docx]

**Table S4. Harmonization process of individual educational level of health surveys across countries**

|  | **Questions** | **Responses included in each category according to IPUMS Criteria** | | | |
| --- | --- | --- | --- | --- | --- |
|  |  | **Less than Primary** | **Primary** | **Secondary** | **University** |
| **Mexico** | Question 1 (S1Q1). “What is the last year/grade you completed?” Response options are numeric values Question 2 (S1Q2). “What level of education was that year/grade in?” | Never attended (0y), Pre-school, Primary (1-5 years) | Primary education (≥6 years), Secondary, Preparatory (1-2 years), Preparatory or Bachelor Basic School, Technical commercial with primary complete, Technical commercial with secondary complete (1-2 years) | Secondary, Preparatory (≥3 years), Technical commercial with secondary complete (≥3 years), Teacher's training, University or Professional (1-3 years) | University or Professional (≥4 years), Masters, Doctorate |
| **El Salvador** |  | Never attended (0y), Pre-school, Primary or basic (1-5 years) | Primary education (≥6 years), Secondary education (1-2 years) | Secondary, Preparatory (≥3 years), Technical Education program, non-university specialized institution, Higher/College Education (1-4 years) | University or Professional (≥5 years), Masters, postgraduate or doctoral studies |
| **Chile** |  | Never attended (0y), Pre-school, Special education, Primary or preparatory (1-5 years) | Primary education (≥6 years), Humanities (Old system, 1-5 years), Secondary education (1-3 years), Technical (Old system, 1-4 years), Secondary or Technical Professional (1-3 years) | Humanities (Old system, ≥6 years), Secondary education-Technical (≥4 years), Technical (Old system, ≥5 years), Secondary-Technical Professional ( ≥4 years) years), Professional Institue, Professional careers (1-3 years) | Professional career (≥4 years), Postgraduate |
| **Guatemala** | Question 1 (S2Q1). “What was the highest level of education you earned?” Responses are numeric values | <6 years of education | 6-11 years of education | 12-16 years of education | ≥17 years of education |
| **Peru** |  | 0-5 years of education | 6-10 years of education | 11-15 years of education | ≥16 years of education |
| **Argentina** | This is a constructed variable “Level of Education” from the original survey. The original variables from which it was created determined if the respondent had ever attended school, the highest level of education entered, if that level was completed, and what the highest grade completed was if the level was not completed | Never attended (0y), Primary incomplete | Primary complete, Secondary incomplete | Secondary complete, Tertiary or University incomplete | Tertiary or University complete |
| **Brazil** | Question 9 (S4Q9). “What was the highest level of schooling last attended?”. Question 13 (S4Q13). “What was the last year/grade completed with a passing grade in this level that was previously attended?”. Question 14 (S4Q14). “Was this level last attended completed?” | Alphabetization course, Pre-school, Primary-elementary (1-4 years), Primary school (1-4 years), Middle school (1-4 years), Supplemental primary education (1-4 years) | Middle school (≥5 years), Primary-elementary (Pre 1971, ≥ 5 years), Middle School (≥ 5 years), Primary school (≥ 5 years), Supplemental primary education (≥ 5 years), High school (Pre-1971, 1-2 years), Secondary school (1-2 years), Supplemental secondary education (1-2 years) | High school (Pre-1971, ≥3 years), Secondary school (≥3 years), Supplemental secondary education (≥ 3 years), Post-Secondary (College, 1 year or 2-6 years incomplete) | Post-Secondary (College, 2-6 years completed), Masters, Doctorate |
| **Colombia** | Question 1 (S7Q1). “Did you ever attend school?” Yes – Go to S7Q2 No – End Question 2 (S7Q2). “What is the highest grade you attended?” Question 3 (S7Q3). “What is the last year/grade you completed?” Response options are numeric values | Never attended (0y), Pre-school, Primary (0-4 years) | Primary education (≥5 years), Secondary or middle education (0-10 years) | Secondary or middle (11-13 years), Technical or Technological with title, Technical or Technological with no title, University with no title | University with title, Masters with title, Masters with no title, Doctorate with title, Doctorate with no title |
