# Supplementary material for: Association of socio-economic environment and women’s empowerment with daily fruit and vegetable intake in Latin American cities: a multilevel study
Source: BMC Public Health. 2025 Jul 2;25:2189. doi: 10.1186/s12889-025-22973-0 (PMC12219996; doi:10.1186/s12889-025-22973-0)
Supplement: Supplementary file 5 — Supplementary Material 5. [file 12889_2025_22973_MOESM5_ESM.docx]

**Table S5. Gender-stratified prevalence ratios of F&V daily intake associated with city variables by individual-level education.**

|  | **Individual-level education** | | | |  |
| --- | --- | --- | --- | --- | --- |
|  | **Less than primary PR (95% IC)** | **Primary PR (95% IC)** | **Secondary PR (95% IC)** | **University PR (95% IC)** | **Global P** |
| **Women** |  |  |  |  |  |
| **GDP per capita** |  |  |  |  |  |
| Tertile 3 vs. Tertile 1 | 1.15 (1.05; 1.24)* | 1.06 (0.98; 1.13) | 1.04 (0.98; 1.11) | 1.02 (0.96; 1.08) | 0.010 |
| **Women’s Empowerment** |  |  |  |  |  |
| Z-Score, each 1 SD increase | 1.21 (1.14; 1.30)** | 1.12 (1.06; 1.18)** | 1.11 (1.04; 1.17)* | 1.08 (1.02; 1.15)* | <0.001 |
| **Living conditions score** |  |  |  |  |  |
| Z-Score, each 1 SD increase | 1.18 (1.11; 1.26)** | 1.10 (1.05; 1.15)** | 1.08 (1.03; 1.13)* | 1.05 (1.00; 1.09)* | <0.001 |
| **Men** |  |  |  |  |  |
| **GDP per capita** |  |  |  |  |  |
| Tertile 3 vs. Tertile 1 | 1.16 (1.05; 1.24)* | 1.04 (0.97; 1.14) | 1.06 (0.98; 1.14) | 1.09 (1.00; 1.18)* | 0.015 |
| **Women’s Empowerment** |  |  |  |  |  |
| Z-Score, each 1 SD increase | 1.13 (1.05; 1.23)* | 1.12 (1.04; 1.20)* | 1.11 (1.04; 1.20)* | 1.10 (1.02; 1.19)* | 0.636 |
| **Living conditions score** |  |  |  |  |  |
| Z-Score, each 1 SD increase | 1.19 (1.11; 1.28)** | 1.10 (1.04; 1.17)* | 1.07 (1.01; 1.14)* | 1.06 (1.00; 1.12)* | <0.001 |

PR: Prevalence Ratio; CI: Confidence Interval. City per capita GDP (Gross Domestic Product) expressed in 2011 USD power purchase parity (ppp); SD: Standard Deviation. *p<0.05; **p<0.001. Model 1 is adjusted by country. Model 2 is adjusted by country, gender, age, individual educational level, GDP per capita, climate zone, city size and city educational attainment (Z-score).
