# Supplementary material for: Association of socio-economic environment and women’s empowerment with daily fruit and vegetable intake in Latin American cities: a multilevel study
Source: BMC Public Health. 2025 Jul 2;25:2189. doi: 10.1186/s12889-025-22973-0 (PMC12219996; doi:10.1186/s12889-025-22973-0)
Supplement: Supplementary file 6 — Supplementary Material 6. [file 12889_2025_22973_MOESM6_ESM.docx]

**Table S6. Gender-stratified prevalence ratios of daily intake of fruits or vegetables associated with social environment variables**

|  | Daily intake of fruits | | Daily intake of vegetables | |
| --- | --- | --- | --- | --- |
|  | **Model 1 PR (95% CI)** | **Model 2 PR (95% CI)** | **Model 1 PR (95% CI)** | **Model 2 PR (95% CI)** |
| Women |  |  |  |  |
| GDP per capita |  |  |  |  |
| Tertile 1 (<$11,100 USD ppp) | 1.00 (Reference) | 1.00 (Reference) | 1.00 (Reference) | 1.00 (Reference) |
| Tertile 2 ($11,100 to $16,200 USD ppp) | 1.26 (1.06, 1.27)* | 1.12 (1.03, 1.22)* | 1.12 (0.95, 1.31) | 1.05 (0.92, 1.21) |
| Tertile 3 (>$16,200 USD ppp) | 1.20 (1.11, 1.32)** | 1.17 (1.07, 1.28)* | 1.07 (0.93, 1.24) | 1.11 (0.97, 1.27) |
| Women’s Empowerment |  |  |  |  |
| Z-Score, each 1 SD increase | 1.12 (1.05, 1.20)* | 1.08 (0.99, 1.17) | 1.21 (1.09, 1.33)** | 1.24 (1.10, 1.39)** |
| Living conditions score |  |  |  |  |
| Z-Score, each 1 SD increase | 1.17 (1.10, 1.23)** | 1.15 (1.07, 1.23)** | 1.12 (1.05, 1.22)* | 1.11 (1.02, 1.20)* |
| Men |  |  |  |  |
| GDP per capita |  |  |  |  |
| Tertile 1 (<$11,100 USD ppp) | 1.00 (Reference) | 1.00 (Reference) | 1.00 (Reference) | 1.00 (Reference) |
| Tertile 2 ($11,100 to $16,200 USD pp) | 1.15 (1.03, 1.29)* | 1.13 (1.02, 1.25)* | 1.06 (0.93, 1.21) | 1.12 (0.95, 1.31) |
| Tertile 3 (>$16,200 USD ppp) | 1.16 (1.04, 1.29)* | 1.13 (1.01, 1.26)* | 1.11 (0.98, 1.25) | 1.09 (0.93, 1.28) |
| Women’s Empowerment |  |  |  |  |
| Z-Score, each 1 SD increase | 1.03 (0.94, 1.13) | 1.00 (0.89, 1.13) | 1.14 (1.01, 1.28)* | 1.17 (1.02, 1.33)* |
| Living conditions score |  |  |  |  |
| Z-Score, each 1 SD increase | 1.14 (1.04, 1.25)** | 1.15 (1.04, 1.28)* | 1.10 (1.01, 1.19)* | 1.08 (0.99, 1.19) |

PR: Prevalence Ratio; CI: Confidence Interval. City per capita GDP (Gross Domestic Product) expressed in 2011 USD power purchase parity (ppp); SD: Standard Deviation. *p<0.05; **p<0.001. Model 1 is adjusted by country. Model 2 is adjusted by country, gender, age, individual educational level, GDP per capita, climate zone, city size and city educational attainment (Z-score).
