# Supplementary material for: Association of socio-economic environment and women’s empowerment with daily fruit and vegetable intake in Latin American cities: a multilevel study
Source: BMC Public Health. 2025 Jul 2;25:2189. doi: 10.1186/s12889-025-22973-0 (PMC12219996; doi:10.1186/s12889-025-22973-0)
Supplement: Supplementary file 7 — Supplementary Material 7. [file 12889_2025_22973_MOESM7_ESM.docx]

**Table S7. Gender-stratified prevalence ratios of daily intake of fruits associated with social environment variables by idividual-level education.**

|  | **Individual-level education** | | | |  |
| --- | --- | --- | --- | --- | --- |
|  | **Less than primary PR (95% IC)** | **Primary PR (95% IC)** | **Secondary PR (95% IC)** | **University PR (95% IC)** | **Global P** |
| **Women** |  |  |  |  |  |
| **GDP per capita** |  |  |  |  |  |
| Tertile 3 vs. Tertile 1 | 1.31 (1.16; 1.38) | 1.18 (1.06; 1.32) | 1.15 (1.04; 1.26) | 1.08 (0.98; 1.20) | 0.010 |
| **Women’s Empowerment** |  |  |  |  |  |
| Z-Score, each 1 SD increase | 1.17 (1.07; 1.28) | 1.07 (0.99; 1.17) | 1.11 (1.02; 1.21) | 1.10 (1.01; 1.21) | 0.031 |
| **Living conditions score** |  |  |  |  |  |
| Z-Score, each 1 SD increase | 1.19 (1.09; 1.30) | 1.12 (1.04; 1.20) | 1.16 (1.08; 1.24) | 1.17 (1.09; 1.25) | 0.047 |
| **Men** |  |  |  |  |  |
| **GDP per capita** |  |  |  |  |  |
| Tertile 3 vs. Tertile 1 | 1.31 (1.11; 1.55) | 1.11 (0.97; 1.26) | 1.12 (0.99; 1.26) | 1.09 (0.95; 1.24) | 0.016 |
| **Women’s Empowerment** |  |  |  |  |  |
| Z-Score, each 1 SD increase | 1.03 (0.90; 1.18) | 1.01 (0.90; 1.13) | 1.02 (1.01; 1.15) | 1.07 (0.94; 1.22) | 0.353 |
| **Living conditions score** |  |  |  |  |  |
| Z-Score, each 1 SD increase | 1.26 (1.11; 1.44) | 1.12 (1.00; 1.25) | 1.11 (1.00; 1.23) | 1.23 (1.11; 1.36) | 0.005 |

PR: Prevalence Ratio; CI: Confidence Interval. City per capita GDP (Gross Domestic Product) expressed in 2011 USD power purchase parity (ppp); SD: Standard Deviation. Models are adjusted by country, gender, age, individual educational level, GDP per capita, climate zone, city size and city educational attainment (Z-score).
