# Supplementary material for: Association of socio-economic environment and women’s empowerment with daily fruit and vegetable intake in Latin American cities: a multilevel study
Source: BMC Public Health. 2025 Jul 2;25:2189. doi: 10.1186/s12889-025-22973-0 (PMC12219996; doi:10.1186/s12889-025-22973-0)
Supplement: Supplementary file 8 — Supplementary Material 8. [file 12889_2025_22973_MOESM8_ESM.docx]

**Table S8. Gender-stratified prevalence ratios of daily intake of vegetables associated with social environment variables by individual-level education.**

|  | **Individual-level education** | | | |  |
| --- | --- | --- | --- | --- | --- |
|  | **Less than primary PR (95% IC)** | **Primary PR (95% IC)** | **Secondary PR (95% IC)** | **University PR (95% IC)** | **Global P** |
| **Women** |  |  |  |  |  |
| **GDP per capita** |  |  |  |  |  |
| Tertile 3 vs. Tertile 1 | 1.21 (1.02; 1.44) | 1.11 (0.95; 1.29) | 1.05 (0.92; 1.21) | 1.14 (1.01; 1.30) | 0.037 |
| **Women’s Empowerment** |  |  |  |  |  |
| Z-Score, each 1 SD increase | 1.35 (1.19; 1.53) | 1.23 (1.10; 1.38) | 1.23 (1.10; 1.39) | 1.24 (1.11; 1.40) | 0.004 |
| **Living conditions score** |  |  |  |  |  |
| Z-Score, each 1 SD increase | 1.29 (1.16; 1.43) | 1.16 (1.04; 1.28) | 1.13 (1.02; 1.24) | 1.12 (1.02; 1.23) | <0.001 |
| **Men** |  |  |  |  |  |
| **GDP per capita** |  |  |  |  |  |
| Tertile 3 vs. Tertile 1 | 1.15 (0.95; 1.41) | 1.09 (0.91; 1.30) | 1.04 (0.89; 1.23) | 1.12 (0.94; 1.34) | 0.173 |
| **Women’s Empowerment** |  |  |  |  |  |
| Z-Score, each 1 SD increase | 1.21 (1.05; 1.40) | 1.19 (1.04; 1.35) | 1.18 (1.04; 1.35) | 1.18 (1.02; 1.35) | 0.929 |
| **Living conditions score** |  |  |  |  |  |
| Z-Score, each 1 SD increase | 1.25 (1.12; 1.41) | 1.15 (1.02; 1.29) | 1.10 (0.98; 1.22) | 1.08 (0.96; 1.22) | <0.001 |

PR: Prevalence Ratio; CI: Confidence Interval. City per capita GDP (Gross Domestic Product) expressed in 2011 USD power purchase parity (ppp); SD: Standard Deviation. Models are adjusted by country, gender, age, individual educational level, GDP per capita, climate zone, city size and city educational attainment (Z-score).
