# Supplementary material for: Association of socio-economic environment and women’s empowerment with daily fruit and vegetable intake in Latin American cities: a multilevel study
Source: BMC Public Health. 2025 Jul 2;25:2189. doi: 10.1186/s12889-025-22973-0 (PMC12219996; doi:10.1186/s12889-025-22973-0)
Supplement: Supplementary file 9 — Supplementary Material 9. [file 12889_2025_22973_MOESM9_ESM.docx]

**Table S9. Comparison of participant’s characteristics with complete data versus those excluded from the analyses**

|  | **Sample with complete data**  **N=91,977** | **Missing data for F&V**  **N=3,470** | **P value** |
| --- | --- | --- | --- |
| **Age, years** | 40 (29; 55) | 38 (28; 51) | <0.0001 |
| **Gender** |  |  |  |
| Women, n (%) | 53.433 (58,1%) | 1.608 (46,3%) | <0.0001 |
| Men, n (%) | 38.544 (41,9%) | 1862 (53,7%) |  |
| **Educational level** |  |  |  |
| Missing values, n (%) | 106 (0,1%) | 0 (0%) | 0.045 |
| Less than primary, n (%) | 16.022 (17,4%) | 711 (20.5%) | <0.0001 |
| Primary, n (%) | 28.993(31,5%) | 865 (24.9%) | <0.0001 |
| Secondary, n (%) | 34.068 (37,0%) | 1.353 (39,0%) | 0.019 |
| University, n (%) | 12.788 (13,9%) | 541 (15,6%) | 0.005 |
| **Characteristics of the city of residence** |  |  |  |
| City size (persons per 100,000) | 11.1 (4.3; 35.7) | 21.7 (8.9; 36.6) | <0.0001 |
| WE, Z-Score | 1.06 (0.49; 1.45) | 1.39 (1.05; 1.64) | <0.0001 |
| GDP per capita, USD 2010 ppp | 14,487 (9,429; 22,036) | 11,071 (9,095; 21,264) | <0.0001 |
| Living conditions score, Z-Score | 0.76 (0.07; 1.18) | 0.91 (0.14; 1.21) | <0.0001 |
| **Climate Zone** |  |  |  |
| Tropical, n (%) | 38,420 (41.8%) | 2,743 (70.1%) | <0.0001 |
| Arid, n (%) | 20,497 (22.3%) | 40 (1.2%) | <0.0001 |
| Template, n (%) | 31,704 (34.5%) | 684 (19.7%) | <0.0001 |
| Polar, n (%) | 1,356 (1.5%) | 3 (0.1%) | <0.0001 |
| **Country** |  |  |  |
| Argentina, n (%) | 21,261 (23.1%) | 190 (5.5%) | <0.0001 |
| Brazil, n (%) | 37,621 (40.9%) | 3,082 (88.8%) | <0.0001 |
| Chile, n (%) | 3,805 (4.1%) | 0 (0%) | <0.0001 |
| Colombia, n (%) | 4,433 (4.8%) | 181 (5.2%) | 0.285 |
| Guatemala, n (%) | 1,396 (1.5%) | 1 (0.0%) | <0.0001 |
| Mexico, n (%) | 10,002 (10.9%) | 0 (0%) | <0.0001 |
| Perú, n (%) | 11,920 (13.0%) | 9 (0.3%) | <0.0001 |
| El Salvador, n (%) | 1,539 (1.7%) | 7 (0.2%) | <0.0001 |
